# The Assessment of the Nutritional Status among the Young-Old and Old-Old Population with Alimentary-Dependent Diseases

**DOI:** 10.3390/medicina60060923

**Published:** 2024-06-01

**Authors:** Gulmira Zhanalina, Svetlana Plyasovskaya, Xeniya Mkhitaryan, Vilen Molotov-Luchanskiy, Vaiva Hendrixson, Zhanerke Bolatova, Zhuldyz Aldanova, Gaukhar Kayupova

**Affiliations:** 1 School of Public Health, Karaganda Medical University, 40 Gogol Street, Karaganda 100008, Kazakhstan; janalina1972@mail.ru (G.Z.); aldanova_z@mail.ru (Z.A.); kayupovag@qmu.kz (G.K.); 2 Department of Physiology, Karaganda Medical University, 40 Gogol Street, Karaganda 100008, Kazakhstan; kseniyamkhitaryan@mail.ru; 3 Department of Internal Medicine, Karaganda Medical University, 40 Gogol Street, Karaganda 100008, Kazakhstan; molotov-luchanskiy@qmu.kz; 4 Faculty of Medicine, Vilnius University, M. K. Čiurlionio g. 21, LT-03101 Vilnius, Lithuania; vaiva.hendrixson@mf.vu.lt

**Keywords:** actual nutrition, nutritional status, alimentary-dependent diseases, proteins, fats, carbohydrates

## Abstract

*Background and Objectives*: Unhealthy nutrition can contribute to the development or progression of various alimentary-dependent diseases, including obesity, type 2 diabetes mellitus, metabolic syndrome, anaemia, and arterial hypertension. Young-old and old-old individuals often have diets deficient in essential vitamins, minerals, and macronutrients, characterized by high consumption of carbohydrate-rich foods and insufficient intake of plant-based products like vegetables and fruits. This study aims to identify key parameters of nutritional status among the young-old (aged 60–74 years) and old-old (aged 75–90 years) populations in central Kazakhstan, particularly in relation to specific alimentary-dependent diseases. *Materials and Methods*: The study involved 300 participants aged 60−90 years. The study incorporated a dietary questionnaire, food consumption records (such as 24 h recalls), and measurements of anthropometric indicators including weight and skinfold measurements. *Results*: Residents in the surveyed regions typically consumed food 3–4 times daily, with breakfast, lunch, and dinner being eaten at consistent times. A significant proportion of individuals, especially older adults, followed this meal schedule. About one-third ate before bedtime, and more than half believed they adhere to a specific diet. The mean BMI for men aged 60–74 years was 28.3 (95% CI: 20.1–43.2) and, for those aged 75–90 years, it was 29.0 (95% CI: 22.1–40.8). Caloric intake among individuals aged 60–74 was higher compared to those aged 75–90, with males consuming an average of 2372.7 kcal and females consuming 2236.78 kcal versus 2101.5 kcal for males and 2099.9 kcal for females in the older age group. *Conclusions*: The dietary patterns observed among old-old individuals were marked by excessive calorie intakes and imbalances in macronutrient composition, with a predominant emphasis on high-carbohydrate foods at the expense of essential nutrients like proteins, fats, and key vitamins (such as C, E, B vitamins) and minerals (such as potassium, calcium, and iron).

## 1. Introduction

Rational nutrition and wholesome foods are significant environmental factors that have direct impacts on the health and life expectancy of older people. Leading a healthy lifestyle, including a healthy diet, is recommended in order to positively influence the existing chronic processes. Compliance with the recommended rules and norms of rational nutrition, in turn, is known to be beneficial for general health [1,2]. The diet of young-old and old-old people often should be adjusted in order to slow the progression of existing alimentary-dependent diseases, as well as for the prevention of such illnesses [3,4]. Unbalanced and unhealthy nutrition is known to affect nutritional status, which may trigger the mechanisms of development or progression of alimentary-dependent diseases, such as obesity, type 2 diabetes mellitus, metabolic syndrome, anaemia, or arterial hypertension [5,6,7,8].

According to the World Health Organization (WHO), approximately 1.28 billion adults aged 30–79 years worldwide have hypertension, with the majority (two-thirds) living in low- or middle-income countries [9]. In Kazakhstan, the prevalence of arterial hypertension in the general population ranges from 15.2% to 27%, and, among women, it is around 40%, as reported by various sources [10]. Moreover, epidemiological studies conducted by Alikhanova et al. reveal that the predominant health issues affecting the adult population are related to circulatory system diseases, characterised by elevated blood pressure [11].

Moreover, the study by Galiyeva et al. emphasised the prevalence of type 2 diabetes in Kazakhstan, with a reported rate of 2075 cases per 100,000 of the population in 2019 [12]. This significant prevalence of type 2 diabetes represents a substantial burden on the healthcare system in Kazakhstan [13].

Obesity is increasingly recognized as a significant public health concern affecting the global population [14]. According to the WHO, in 2022, the prevalence of obesity affected one in eight individuals globally. Since 1990, the rates of adult obesity worldwide have more than doubled, while adolescent obesity has quadrupled [15].

The diet of older individuals is frequently characterized by deficiencies in essential vitamins, minerals, and macronutrients, alongside high consumption of carbohydrate-rich foods and inadequate intake of plant products like vegetables and fruits. Kerstetter et al. emphasised that older individuals are less efficient at absorbing calcium from low or marginal intakes compared to when they were younger, indicating a potential risk of calcium deficiency in older adults with insufficient dietary calcium intake [16]. Chen et al. conducted a study on the prevalence of common micronutrient deficiencies in China and found that inadequate diets, particularly in households of a lower socio-economic status, increase the risk of multiple concurrent micronutrient deficiencies, highlighting a troubling trend of inadequate nutrient intake in certain populations [17]. This nutrient imbalance can contribute to the development or exacerbation of various diet-related diseases, including cardiovascular issues, endocrine disorders, blood and gastrointestinal problems, and even cancer. Lorenzo-López et al. suggested that poor nutritional status significantly influences frailty in older adults [18]. Frailty, a geriatric syndrome associated with disability, morbidity, and mortality, is closely linked to inadequate nutrition and micronutrient deficiencies [19]. Protein deficiencies, particularly in older individuals, can lead to declines in skeletal muscle mass and strength, contributing to increased mortality and reduced quality of life [20]. Adequate protein intake is essential for maintaining muscle health and overall wellbeing in the old-old population. Moreover, deficiencies in vitamins and minerals, such as vitamin D, B12, and folic acid, are prevalent among older individuals [21]. These deficiencies can have far-reaching health implications, emphasising the importance of addressing nutrient imbalances in the diet of older adults. While a healthy diet is important for people of all ages, it is important to focus on the nutrition of the older population due to the specifics of ageing metabolisms, the functionality of organs and systems, and their eating habits [8,22,23]. Rational and balanced nutrition in the old and old-old affects the ageing process and the functional deviations inherent in the organs and systems of the body [23,24]. On the opposite side, an unbalanced diet and malnutrition activate the mechanisms of premature ageing, which subsequently lead to disability and death. Active longevity is a norm of modern society, and the nutritional status and habits of the old-old population are important factors in regard to meeting the needs of the old population [25]; however, such research seems to be lacking in Kazakhstan. This gap is significant, as the young-old and old-old individuals are particularly vulnerable to malnutrition, which can exacerbate chronic conditions like hypertension, diabetes, and obesity. Understanding their nutritional status is crucial for developing targeted interventions to improve health outcomes. This study aims to fill this gap by providing comprehensive data on the dietary patterns and nutritional status of the young-old and old-old individuals in Kazakhstan, informing public health strategies and policies to enhance their wellbeing.

A comprehensive nutritional status assessment encompasses a thorough clinical examination, which includes gathering detailed medical history and conducting a physical examination to identify signs of malnutrition or specific nutrient deficiencies [26]. In line with this, our study aims to determine the characteristic parameters of the nutritional statuses of the young-old (60 to 74 years of age) and old-old (75–90 years) populations in central Kazakhstan in regard to certain alimentary-dependent diseases. The study is focused on the three most common issues: obesity, hypertension, and type 2 diabetes mellitus.

## 2. Materials and Methods

### 2.1. Sample Size and Data Collection

The sample size of 300 study subjects aged 60−90 was achieved by contacting 524 individuals from the target population of seniors with prevalent chronic conditions, such as obesity, hypertension, and type 2 diabetes. For the study, 286 participants (143 males and 143 females) were required to achieve an 80% power to detect a sex difference in the frequency of occurrence of a trait, with a 5% significance level, using the Z-test for proportions when the population size was unknown. The sample size was increased to 300 participants to account for potential attrition [27]. The contacted individuals were screened for eligibility based on age and health criteria, resulting in the recruitment of 300 participants for the study. A total of 21.6% of them were male (*n* = 65), whereas 78.3% were women (*n* = 235). Among the respondents, the following groups were identified as young-old (60–74 years of age) and old-old (75–90 years of age) with a history of alimentary-related non-communicable diseases.

Data were collected from four primary healthcare centres of the Karaganda region (central Kazakhstan). Patients with registered alimentary-dependent morbidities were invited for routine checkups by an endocrinologist. Alimentary-related non-communicable diseases included obesity, hypertension, and type 2 diabetes mellitus. After the appointment, the specialist referred patients to the researcher according to the inclusion criteria of the study. The researcher explained the purpose of the study and that it was voluntary. Study subjects read and signed the consent form. The informed consent process involved providing participants with detailed information about the study, including its purpose, procedures, benefits, and their rights as participants. Participants were given ample time to review the information and ask any questions they may have had before voluntarily deciding whether to participate. Written consent was obtained from each participant who agreed to take part in the study.

As the study participants were old-old, the questionnaire items were read to each respondent by the researcher, who then facilitated the selection of the appropriate answer option. Additionally, all measurements were conducted by the researcher. The duration of each study session ranged from approximately 30 to 40 min per participant. The completion time for the questionnaire ranged from 12 to 15 min per participant.

Ethical approval of the study was obtained from the Bioethics Committee of Karaganda Medical University” NCJSC on 17 June 2019 (Meeting No. 20, Protocol No. 61). The research was conducted according to relevant guidelines and regulations.

### 2.2. Study Design

The study was conducted based on the program CINDI (WHO) “Countrywide Integrated Non-communicable Diseases Intervention (CINDI) Programme” [28]. The primary goal of CINDI was to enhance the wellbeing of communities by decreasing the occurrence of morbidity and mortality caused by significant non-communicable diseases (NCDs) through a comprehensive and cooperative intervention program that focuses on prevention and the promotion of health. The study included a dietary questionnaire, food consumption records or 24 h recalls, and measurement of anthropometric indicators, including weight and skinfold measurements.

### 2.3. Measurements

Respondents’ weights were assessed by Tanita Body Composition Analyzer SC-330, which performs a bioelectrical analysis of the entire body, whereas measurement of their subscapular, triceps, biceps, and abdominal skinfolds was assessed with calipometry. Skin folds were measured on the right side of the torso using a calipometry. The skin fold on the right side of the torso should be squeezed between the thumb and index finger while applying the calipometry feet, which should be then applied horizontally to the desired points without pressure (an average of three measurements were taken).

The questionnaire contained sections such as socio-demographic indicators, awareness of healthy eating habits, dietary habits, and food consumption records. A pilot version of the questionnaire was used in Kazakhstan on 8219 people aged 18−73 years (37% men, 63% women) [29]. Socio-demographic indicators included place of origin, date of birth, gender, nationality, marital status, education, income level, and number of individuals in the family. Moreover, the awareness of healthy eating habits sections had questions regarding daily consumption of fresh fruits and vegetables; predominant foods in the daily diet with different food categories, including fats and sweets, meat and fish, milk and dairy products, vegetables and fruits, cereals, and bread; opinions on the healthiest type of bread and meat; opinions about pasta (healthy/not); views on the nutritional value of potatoes; availability of milk, with varying fat contents in-store; perception of the healthiest milk choice; preference for a specific type of salt; preferred cooking fat for health benefits; and sources of nutrition information.

Furthermore, the dietary habits part of the questionnaire contained information about the frequency of daily meals; dietary preferences; breakfast habits; lunch habits; dinner habits; additional meals; late-night eating; consumption of hot meals; cooking fat preferences (in meals and sandwiches); preference in milk; salt usage and its type; bread choices; kind of meat consumption; dairy products (milk, kefir, ayran, yoghurt); cheese and types; butter; fat and type of oil usage; sausage products; canned meat and fish; fish and seafood; eggs; grain products; vegetables; fruits and berries; beverages and drinks. In addition, participants were asked about their daily food diary, with items included the foods they consumed yesterday and their reasons for eating them, doctor’s recommendations, specialized diet details, and religious practices.

### 2.4. Statistical Analysis

Statistical data processing was conducted using Statistica 8.0 for Windows. The statistical significance of differences was assessed using both parametric and non-parametric statistical methods. Comparative analysis of independent samples with a normal distribution was carried out using the Student’s *t*-test. Comparative analysis of independent samples with a distribution different from normal was conducted using the Mann–Whitney U test. Comparative assessment of qualitative characteristics was performed using the chi-square test and the Student’s *t*-test for proportions, with the calculation of confidence intervals.

## 3. Results

### 3.1. Dietary Habits

The dietary behaviour of residents in the studied regions was characterized by the following features: a large portion of respondents consumed food 3–4 times a day 58–60% men; 70–75% women. They had breakfast, lunch, and dinner at the same time, with women 75–90 years at 65%, women 60–74 years at 71%, men 75–90 years at 80%, and men 60–74 years at 74%. One-third of the respondents in the surveyed localities consumed food before bedtime. Moreover, more than half of the respondents believed that they adhere to a diet. Hot food (cooked meals) was consumed twice a day by a large part of the respondents (57–58% women, 70% men). About one-sixth of the respondents consumed hot food once a day.

Women 60–74 years and 75–90 years consumed first courses daily, at 13% and 27%, respectively, with men being at 6% and 20%, with 32–34% of the respondents consuming hot food four times a week or more. Women consumed first courses no more than three times a week (45–47%), with men being at 60%, while the remaining 4% of respondents did not consume first courses at all.

Flour-based food emerged as the leader in food preferences, with 63% of women aged 60–74 years, 51% of women aged 75–90 years, 48% of men aged 60–74 years, and 33% of men aged 75–90 years preferring it. The second priority food product for the population of the surveyed regions was meat for men aged 60–74 years at 38% and men aged 75–90 years at 27%, and plant-based food for women aged 60–74 years in 24% of cases and women aged 75–90 years in 37% of cases. This was followed by plant-based food for men aged 60–74 years at 14% and men aged 75–90 years at 40%, meat for women aged 60–74 years at 11%, and women aged 75–90 years at 7% of cases. Regarding salty food, the respondents’ opinions were distributed as follows: in men aged 60–74 years and 75–90 years, 70% and 66% liked salty food, while in women aged 60–74 years and 75–90 years it was 49% and 45%. For making sandwiches, butter was preferred by men aged 60–74 years and 75–90 years at 78% and 80%, while in women aged 60–74 years and 75–90 years, it was at 76% and 74%.

Young-old and old-old individuals often consumed milk, meat, butter, pasta and cereal products, confectionery and bakery products, sugar, and tea more frequently. They more commonly used vegetable oil for cooking. They significantly less often consumed fermented milk products, cottage cheese and cheese, vegetables and fruits, juices, meat byproducts, fish, eggs, meat, and fish canned goods.

### 3.2. Anthropometric Characteristics

The average height for men aged 60–74 was 170.7 cm, with a confidence interval ranging from 155 to 189 cm, whereas the average height for men of 75–90 years was 167 cm (95% CI = 156.0–186.0). There was no statistically significant difference in the height of these two groups with a *p*-value of 0.167. Moreover, the mean weight for old-old men was 82.6 kg (95% CI = 56.0–122.0), and for young-old men it was 74.7 with 95% CI varying from 0 to 111.0 kg, without a statistically significant difference of 0.307. The mean BMI for men aged 60–74 years was 28.3, with a 95% CI of 20.1–43.2, while for those aged 75–90 years, it was 29.0, with a 95% CI of 22.1–40.8 (*p* = 0.708) (Table 1).

Skinfold measures at various sites also showed no significant differences between the two age groups, with *p*-values ranging from 0.286 to 0.981. Men aged 60–74 years had a mean of subscapular skinfold measure of 27.7 mm (95% CI: 16.0–46.0 mm) compared to 27.8 mm (95% CI: 17.0–48.0 mm) for those aged 75–90 years (*p* = 0.981). Similarly, the triceps skinfold measures showed a mean of 26.1 mm (95% CI: 12.0–46.0 mm) for the old-old group and 29.3 mm (95% CI: 14.0–47.0 mm) for the young-old group (*p* = 0.286). Biceps skinfold measures averaged 27.2 mm, with 95% CI ranging from 12.0 to 45.0 mm and 29.2 mm (95% CI: 18.0–44.0 mm) for men aged 60–74 and 75–90 years, respectively (*p* = 0.469). Lastly, the abdominal skinfolds measured 46.1 mm (95% CI: 15.0–76.0 mm) and 50.1 mm, with 95% CI varying from 21.0–85.0 mm for the two age groups, respectively (*p* = 0.493).

The mean height for females aged 60–74 years was 158.1 cm, with a 95% confidence interval ranging from 148 cm to 176 cm. The similar mean height for females aged 75–90 years was 158.1 cm, with a 95% CI ranging from 147 cm to 175 cm. There was no statistically significant difference in height between the two age groups, with a *p*-value of 0.268. Notable disparities were in weight and BMI. Women aged 75–90 years had a lower mean weight (73.7 kg, 95% CI: 45–106 kg) compared to those aged 60–74 years (79.4 kg, 95% CI: 46–163 kg), with a significant *p*-value of 0.035. Similarly, the BMI for the young-old group (29.5, 95% CI: 20–42.5) was significantly lower than that of the old-old group (31.2, 95% CI: 19.6–50.3), with a *p*-value of 0.005. Subscapular skinfold measurements showed differences between age groups, with a *p*-value of 0.01. The old-old women’s average subscapular skinfold measurement was statistically higher at 29.3 mm, with 95% CI ranging from 7.0 to 98.0 mm, whereas young-old women had a lower subscapular skinfold thickness at 25.4 mm (95%CI = 13.0–43.0 mm). There were no statistically significant differences in triceps, biceps and abdominal skinfold measurements between the two groups, which varied from 0.084 to 0.597.

### 3.3. Nutritional and Biological Value

The average mass of daily dietary intake for old-old males was 1563.0 g, slightly higher than for young-old males, who averaged 1508.3 g. For females, those aged 60–74 consumed an average of 1471.0 g, and those aged 75–90 consumed slightly less, with an average of 1439.7 g. The differences between the age groups for both sexes were not statistically significant, as indicated by *p*-values of 0.539.

Males aged 60–74 had an average water intake from food of 1209.9 mL compared to 1159.4 mL for males aged 75–90. Old-old females had an average intake of 1139.3 mL, with those young-old aged slightly lower at 1126.8 mL. These differences were also not statistically significant (*p*-value = 0.477 for males and 0.478 for females), suggesting similar hydration from food across the studied age ranges for both sexes.

The average total protein intake for males was 55.7 g for those aged 60–74 and 51.8 g for those aged 75–90. Females consumed less protein on average, with those aged 60–74 consuming 48.6 g and those aged 75–90 consuming 45.3 g. The differences in protein intake between the age groups for both sexes were not statistically significant (*p*-value = 0.897) (Table 2).

Males aged 60–74 consumed an average of 61.4 g of fat, which decreased to 49.7 g for those aged 75–90. Females showed a similar pattern, with those aged 60–74 consuming 55.7 g of fat, decreasing to 49.2 g for those aged 75–90. These decreases were not statistically significant, with *p*-values of 0.698 for males and 0.571 for females, suggesting similar fat consumption across these age groups.

Cholesterol intake showed no significant change with age for both sexes. Males consumed an average of 200.4 mg (60–74 age group) and 179.6 mg (75–90 age group), while female intake was 230.1 mg and 217.7 mg, respectively. The *p*-values (0.948 for males and 0.406 for females) suggested stable cholesterol consumption patterns across the studied age ranges.

Intake of mono- and disaccharides was slightly higher in females compared to males, with no significant change being seen with age within each sex. The *p*-values were 0.561 for males and 0.186 for females, indicating stable sugar consumption across these age groups. Starch intake decreased with age in both sexes, from 213.7 g to 192.9 g in males and from 187.9 g to 172.9 g in females. These changes were not statistically significant (*p*-values: 0.698 for males and 0.314 for females).

Both males and females aged 60–74 consumed higher amounts of total carbohydrates compared to their counterparts aged 75–90, with means of 399.3 g and 361.6 g for males and 385.1 g and 368.9 g for females, respectively. However, these differences were not statistically significant (*p*-values: 0.477 for males and 0.830 for females), indicating similar carbohydrate intakes across the age groups.

There was no significant difference in dietary fibre intake between males aged 60–74 and those aged 75–90. However, females aged 60–74 consumed slightly more dietary fibre (13.9 g) compared to those aged 75–90 (10.9 g), with a statistically significant difference indicated by a *p*-value of 0.018.

Both males and females aged 60–74 had higher caloric contents compared to those aged 75–90, with means of 2372.7 kcal and 2101.5 kcal for males and 2236.78 kcal and 2099.9 kcal for females, respectively. However, the differences were not statistically tested (Table 2).

### 3.4. Mineral Substances

Both males and females aged 60–74 had higher sodium intakes compared to their counterparts aged 75–90. The mean sodium intake for old-old males was 2977.4 mg, while for young-old males it was 2418.2 mg. Among old-old females, the average sodium intake was 2522.3 mg, and, for young-old females, it was 2166.2 mg. However, this difference was not statistically significant for males (*p*-value = 0.155), while it was significant for females (*p*-value = 0.003), suggesting a decrease in sodium intake with age, particularly among females (Table 3).

The mean potassium intake for males aged 60–74 was 1779.7 mg, and, for males aged 75–90, it was 1691.14 mg. Among females aged 60–74, the average potassium intake was 1696.5 mg, while for females aged 75–90, it was 1606.1 mg. Statistical analysis revealed no significant differences in potassium intake between the two age groups within each sex (*p*-values > 0.05). Notably, both males and females aged 75–90 tended to have slightly lower potassium intakes compared to those aged 60–74, although these differences did not reach statistical significance.

The mean calcium intake for old-old males was 472.4 mg, and, for young-old males, it was 479.81 mg. Among females aged 60–74, the average calcium intake was 490.8 mg, while for females aged 75–90, it was 508.3 mg. Statistical analysis revealed no significant differences in calcium intake between the two age groups within each sex (*p*-value = 0.301) for males. However, there was a significant difference in calcium intake between the age groups for females (*p*-value = 0.02), indicating a decrease in calcium intake with age among females.

For old-old males, the average magnesium intake was 217.8 mg, while, for young-old males, it was 209.77 mg. Among women aged 60–74, the average magnesium intake was 203.0 mg, and for those aged 75–90, it was 191.7 mg. Notably, there were no statistically significant differences in magnesium intake between the two age groups within each sex (*p*-values > 0.05). However, it is worth mentioning that both males and females aged 75–90 tended to have slightly lower magnesium intakes compared to their counterparts aged 60–74, although these differences did not reach statistical significance.

For males aged 60–74, the average daily phosphorus intake was 925.1 mg, while, for males aged 75–90, it was 868.8 mg. Among females aged 60–74, the average phosphorus intake was 841.3 mg, and for those aged 75–90, it was 807.3 mg. Notably, there were no statistically significant differences in phosphorus intake between the two age groups within each sex (*p*-values > 0.05).

There were no significant differences in iron intake between the two age groups within each sex (*p*-values > 0.05). Both males and females aged 75–90 tended to have slightly lower iron intakes compared to those aged 60–74, but these differences were not statistically significant (Table 3).

### 3.5. Vitamins

The average retinol equivalent intake for old-old males was 1107.5 mg, whereas for young-old males, it was 371.8 mg. Among females aged 60–74, the mean retinol equivalent intake stood at 761.2 mg, while for females aged 75–90, it was 744.4 mg. Notably, both males and females aged 60–74 exhibited significantly higher mean retinol equivalent intakes compared to their counterparts aged 75–90. However, statistical analysis revealed that these differences were not statistically significant (with *p*-values greater than 0.05).

For old-old males, the average intake of tocopherol equivalents was 7.6 mg, while for young-old males, it was 6.8 mg. Among females aged 60–74, the mean intake stood at 7.0 mg, and for females aged 75–90, it was 6.3 mg. There were no significant differences in tocopherol equivalent intake between the two age groups within each sex (with *p*-values > 0.05). Notably, both males and females aged 60–74 tended to have slightly higher mean intakes compared to those aged 75–90, although these differences did not reach statistical significance.

The average daily thiamine intake for both old-old men and women aged 60–74 was 0.6 mg, while for women aged 75–90, it was 0.5 mg. Importantly, there were no statistically significant differences in thiamine intake between the two age groups within each sex (with *p*-values > 0.05).

In the 60–74 age group, males consumed riboflavin at an average of 0.9 mg (CI: 0.6–1.0), while females consumed slightly less at 0.8 mg (CI: 0.5–0.9). In the older age group (75–90 years), both males and females showed a decrease in riboflavin intake, with means of 0.8 mg (CI: 0.4–1.0) for males and 0.8 mg (CI: 0.6–0.9) for females. The *p*-values indicated that the differences observed in riboflavin intake between age groups and genders were not statistically significant.

For the niacin equivalent intakes, in regard to the mean values in the 60–74 age group, males consumed an average of 22.0 niacin equivalents (CI: 14.8–25.1), while females consumed slightly less at 18.6 niacin equivalents (CI: 13.1–22.4). In the older age group (75–90 years), both males and females showed a decrease in niacin equivalent intake, with means of 19.9 (CI: 13.5–25.3) for males and 16.5 (CI: 13.2–21.3) for females. There were no significant differences in niacin equivalent intake between the two age groups within each sex (*p*-values > 0.05).

In the 60–74 age group, males consumed an average of 27.6 mg of ascorbic acid (95% CI: 11.7–38.1), while females consumed a notably higher average of 41.6 mg (95% CI: 13.6–41.7). Conversely, in the older age group (75–90 years), both males and females exhibited a decrease in ascorbic acid intake, with means of 21.7 mg (95% CI: 9.1–29.9) for males and 25.4 mg (95% CI: 10.9–35.5) for females. The chi-square test showed that the differences were not statistically significant (Table 4).

## 4. Discussion

In this study, we delved into the dietary habits, anthropometric characteristics, and nutritional profiles of old-old and young-old individuals, examining both males and females. Our investigation encompassed a comprehensive assessment of various aspects, including the nutritional and biological value of food, mineral substances, and vitamins. Through analysis, we explored the nutritional status, shedding light on potential gender disparities and age trends among the old-old demographic group.

Anthropometric measurements have been utilized in numerous studies to evaluate nutritional risks, such as inadequate protein intake [30]. It is essential not only to assess dietary intakes in terms of macronutrients (calories, protein, and fat) but also to explore their relationship with outcome-focused indicators like anthropometric measurements; [31]. During our study, positive energy value (kcal) was evaluated, and this led to an increase in anthropometric measurements (body weight, skin-fat folds). Moreover, our study revealed that the BMI values observed in all age categories of both genders, except for women aged 60–74, were classified as overweight. However, women aged 60–74 were classified as obese based on their BMI values.

The diet of the surveyed respondents could be characterized as a diet with carbohydrate orientation and an imbalance of vitamin and mineral intake [32,33]. It should be noted that the average daily diet of the interviewed men and women was characterized by extremely rare consumption of canned meat and fish, animal fats, and margarine, which, rather, could be attributed to a positive characteristic of the diet [34]. The absence of legume products was explained by the absence of products in the national cuisines of the majority of the population living in Kazakhstan [35]. The absence of berries, honey, sunflower seeds, nuts and mushrooms was explained either by the absence of the habit of consuming these products from early childhood or by the seasonality of the above products, as well as the absence of coffee and meat byproducts [36]. Meat, sausages, eggs, pasta, flour confectionery, cereals, potatoes, fresh cucumbers and tomatoes, apples and pears, confectionery and tea, which were habitual for the diet of Kazakhstan citizens, could be attributed to the products of high frequency of consumption [37]. Products with a predominant frequency of consumption “not more than once a week” or “several times a month” included fish; sauerkraut; canned vegetables; melons, which could be explained by the seasonality of these crops; citrus fruits; bananas and pineapples; and fruit juices [38,39].

Moreover, in Kazakhstan, there is a regulation on scientifically substantiated physiological norms of food consumption. This regulation specifies the chemical composition and energy value of minimum rational norms of consumption of basic food products for various age groups of male and female populations [40].

For males aged 60–74 years, the recommended protein intake was set at 72.0 g, as per established regulations. However, the observed actual protein intake fell substantially lower, at 16.3 g. Similarly, females within the same age category were advised to consume 63.0 g of protein, yet their actual intake was recorded at a mere 14.4 g. Both genders within the age range of 75–90 years exhibited inadequate dietary protein intake as the recommended intake for females (63 g/day) and males (66 g/day). Actual protein consumption was 45.3 g and 51.8 g, respectively. The notable decline in lean body mass occurred with ageing, particularly among post-menopausal women and individuals over 70 years old [41]. This progressive loss of lean body mass underscores the critical importance of adequate protein intake to mitigate muscle atrophy and decline in functional status, as discussed by Coelho-Júnior et al. in 2018 [42]. Furthermore, Tieland et al. emphasized the significance of not only the quantity but also the quality of protein sources in stimulating muscle protein synthesis and promoting muscle mass gain in old-old individuals [43]. Moreover, the relationship between protein intake and metabolic regulation in older adults with type 2 diabetes was underscored by Huang et al., suggesting that adequate dietary protein intake may contribute to improved metabolic control in this population [44].

Among women aged 60–74 years, the recommended daily fat intake was 63 g, whereas the actual consumption represented 88.5% of the recommended intake. In comparison, men in the same age group had a recommended fat intake of 72 g per day, with the actual consumption averaging 61.4 g per day, accounting for 85.3% of the recommended intake. Among women aged 75–90 years, the recommended fat intake was 60 g per day, while the actual consumption constituted 82% of the recommended intake. Similarly, men in this age group had a recommended fat intake of 66 g per day, but the actual consumption was 49.7 g per day, representing only 75.4% of the recommended intake. However, Liu et.al. indicated that older adults often exceed recommended fat intake levels, which can increase their susceptibility to various disease risks [45].

The recommended carbohydrate intake for males across both age groups was established at 366 g per day. Actual intake surpassed this norm, registering at 109% for individuals aged 60–74 years and 107% for those aged 75–90 years. Conversely, for females within the specified age brackets, the recommended carbohydrate intake stood at 320 g per day for those aged 60–74 years and 305 g per day for those aged 75–90 years. Women of both age cohorts exhibited intakes exceeding the recommended thresholds, reaching 120% of the age-appropriate recommended carbohydrate amounts. Yu et al. emphasised that carbohydrate intake constitutes a substantial proportion of total energy intake in both women and men [46].

The recommended caloric intake was 2400 kcal for men aged 60–74 and 2200 kcal for men aged 75–90. For old-old women, the intake was 2100 kcal, and for young-old women, it was 2000 kcal. Nevertheless, individuals of both genders exceeded the recommended calorie intake across all age groups, with males consuming 111.9% and 110.5% of the recommended amount and females consuming 105.3% and 105.6%, respectively. Wang and Sousa-Poza pointed out that, in China, females aged 50–60 consistently exceed the recommended dietary intake criteria in terms of calorie intake [47]. However, Johnston et al. noted that, in the United States, the mean energy intake by gender among older adults from 2005 to 2010 was comparable to the recommended amounts for sedentary individuals [48].

The elevated sodium intake (men aged 60–74 consuming 229% and men aged 75–90 consuming 201% of the recommended requirement (RR); women aged 60–74 consuming 194% and women aged 75–90 consuming 180% of the RR), increased levels of phosphorus (men aged 60–74 consuming 132% and men aged 75–90 consuming 124% of the RR; women aged 60–74 consuming 120% and women aged 75–90 consuming 115% of the RR), and iron (men aged 60–74 consuming 132% and men aged 75–90 consuming 124% of the RR; women aged 60–74 consuming 111% and women aged 75–90 consuming 67% of the RR) were observed. The elevated intake of sodium, phosphorus, and iron observed among individuals aged 60–74 and 75–90, irrespective of gender, raised notable health concerns. High sodium consumption was associated with an increased risk of diabetic retinopathy in old-old individuals with type 2 diabetes, especially when coupled with low vegetable intake [49]. This underscored the critical need to monitor sodium intake in this age group to mitigate the risk of complications.

Furthermore, there was a moderate excess of magnesium (men aged 60–74 consuming 95% and men aged 75–90 consuming 83% of the RR; women consuming 92–95% of the RR) along with a notable deficiency in potassium intake (men aged 60–74 consuming 35.5% and men aged 75–90 consuming 33.8% of the RR; women aged 60–74 consuming 33.9% and women aged 75–90 consuming 32.1% of the RR) and calcium (men aged 60–74 consuming 40% and men aged 75–90 consuming 36.3% of the RR; women aged 60–74 consuming 38% and women aged 75–90 consuming 42% of the RR). These findings suggested an inadequate consumption of dairy and fermented milk products, fish, fruits, and grains in the daily diet, coupled with excessive consumption of canned products, sausages, and fast food. Magnesium plays a critical role in various physiological functions, and its deficiency can lead to adverse health outcomes, including cellular senescence and increased risk of age-related diseases [50]. Potassium deficiency, prevalent in the old-old population, can significantly impact cardiovascular health. Studies have demonstrated that potassium-enriched diets can reduce cardiovascular mortality and medical expenses among older individuals [51]. Calcium is essential for bone health, and inadequate intake can lead to reduced bone mass and osteoporosis, particularly in older adults [52]. Milk and dairy products serve as significant sources of calcium, contributing to the dietary intake of this mineral in older individuals [53]. Addressing the imbalances in magnesium, potassium, and calcium intake among individuals aged 60–74 and 75–90 is crucial for promoting optimal health outcomes, especially concerning cardiovascular health, bone density, and overall wellbeing.

The vitamin composition of the daily diet among old-old and young-old individuals revealed a state of polyhypovitaminosis across all studied vitamins, encompassing both fat-soluble and water-soluble varieties. The adequacy of daily vitamin intake was characterized by deficiencies in the supply of ascorbic acid, retinol, and tocopherol, as well as vitamins B1 and B2, exceeding 50% in both genders and both ages. However, niacin intake in the diets of the old-old reached an average of 137% of the RR for men aged 60–74 and 124.5% for men aged 75–90. For women, niacin intake was 133.5% for those aged 60–74 and 118.3% for those aged 75–90. Regarding retinol equivalent, intake was at 123% of the RR for men aged 60–74 and 65.9% for men aged 75–90. For women, retinol equivalent intake was 108.7% for those aged 60–74 and 106.3% for those aged 75–90. This could be explained by the presence of a sufficient amount of meat in their diets.

Vitamin E, specifically tocopherol, is essential for maintaining antioxidant balance and promoting healthy aging [54]. Ensuring adequate consumption of vitamin E is crucial for optimizing health outcomes. Additionally, vitamin B1 (thiamine) and vitamin B2 (riboflavin) are vital for various physiological functions, and addressing deficiencies in these B-group vitamins is essential for proper metabolic function and overall wellbeing in the old-old population [55]. Furthermore, deficiencies in ascorbic acid (vitamin C) and retinol (vitamin A) can impair immune function, vision, and overall health [56]. Adequate intake of these vitamins is necessary to support bodily functions and prevent associated health issues.

## 5. Conclusions

Dietary patterns among old-old individuals often exhibit excessive calorie intake and imbalances in macronutrient composition, favouring high-carbohydrate foods over essential nutrients like protein, fats, vitamins, and minerals. To address these issues, interventions should prioritize promoting balanced diets rich in vegetables, fruits, fish, and dairy products while discouraging consumption of high-calorie, high-sodium, and high-sugar processed foods. Implementing nutritional education programs and personalized dietary counselling, as well as encouraging regular physical activity, are crucial strategies for supporting healthier dietary behaviors and enhancing overall wellbeing in the old-old population. A comprehensive approach integrating education and counselling is essential for achieving sustainable improvements in dietary habits and disease prevention among older adults, potentially leading to significant improvements in health and quality of life.

## Figures and Tables

**Table 1:** Anthropometric indicators of respondents.

Indicator	Male (*n* = 65)			Female (*n* = 235)		
	Mean (95%CI) of 60–74 Aged (*n* = 50)	Mean (95%CI) of 75–90 Aged (*n* = 15)	*p*-Value	Mean (95%CI) for 60–74 Aged (*n* = 169)	Mean (95%CI) for 75–90 Aged (*n* = 66)	*p*-Value
Height, cm	170.7 (155.0–189.0)	167 (156.0–186.0)	0.167	158.1 (148.0–176.0)	158.1 (147.0–175.0)	0.268
Weight, kg	82.6 (56.0–122.0)	74.7 (0–111.0)	0.307	79.4 (46.0–163.0)	73.7 (45.0–106.0)	0.035
BMI	28.3 (20.1–43.2)	29.0 (22.1–40.8)	0.708	31.2 (19.6–50.3)	29.5 (20.0–42.5)	0.005
Subscapular skinfold, mm	27.7 (16.0–46.0)	27.8 (17.0–48.0)	0.981	29.3 (7.0–98.0)	25.4 (13.0–43.0)	0.01
Triceps skinfold, mm	26.1 (12.0–46.0)	29.3 (14.0–47.0)	0.286	27.8 (10.0–69.0)	26.5 (14.0–53.0)	0.379
Biceps skinfold, mm	27.2 (12.0–45.0)	29.2 (18.0–44.0)	0.469	28.0 (4.0–105.0)	26.8 (11.0–47.0)	0.597
Abdominal skinfold, mm	46.1 (15.0–76.0)	50.1 (21.0–85.0)	0.493	53.5 (13.0–60.0)	46.6 (17.0–88.0)	0.084

**Table 2:** Nutritional and biological value of respondents.

Indicator	Male			Female		
	Mean (95%CI)		*p*-Value	Mean (95%CI)		*p*-Value
	60–74 Aged	75–90 Aged		60–74 Aged	75–90 Aged	
Mass of daily dietary intake, g	1563.0 (1310.0–1765.0)	1508.3 (1200.0–1770.0)	0.539	1471.0 (1240.0–1675.0)	1439.7 (1240.0–1575.0)	0.539
Water intake from food, mL	1209.9 (1018.0–1379.9)	1159.4 (910.3–1401.2)	0.477	1139.3 (966.6–1298.0)	1126.8 (977.8–1235.8)	0.478
Total protein, g	55.7 (49.8–61.5)	51.8 (43.5–60.1)	0.897	48.6 (35.7–57.1)	45.3 (35.5–54.8)	0.897
Total fat intake, g	61.4 (52.5–70.2)	49.7 (42.4–57.1)	0.698	55.7 (52.1–59.4)	49.2 (44.7–50.8)	0.571
Cholesterol intake, mg	200.4 (113.9–308.1)	179.6 (110.0–296.5)	0.948	230.1 (111.7–275.6)	217.7 (118.2–251.4)	0.406
Mono- and disaccharides, g	183.4 (163.2–203.5)	168.6 (149.7–187.5)	0.561	197.1 (185.85–208.3)	195.7 (181.8–209.6)	0.186
Starch, g	213.7 (190.4–237.1)	192.9 (164.2–221.6)	0.698	187.9 (176.7–199.1)	172.9 (157.4–188.4)	0.314
Total carbohydrates, g	399.3 (363.3–435.2)	361.6 (326.4–396.7)	0.477	385.1 (367.2–402.9)	368.9 (345.5–392.2)	0.830
Dietary fiber, g	12.1 (9.1–16.6)	13.9 (11.7–22.6)	0.518	12.4 (9.6–17.0)	10.9 (8.3–15.4)	0.018
Caloric content, kcal	2372.7 (1736.8–2773.7)	2101.5 (1771.0–2456.5)		2236.78 (2138.7–2334.7)	2099.9 (1975.2–2224.5)	

**Table 3:** Mineral substances of respondents.

Indicator	Male			Female		
	Mean (95%CI)		*p*-Value	Mean (95%CI)		*p*-Value
	60–74 Aged	75–90 Aged		60–74 Aged	75–90 Aged	
Sodium, mg	2977.4 (1798.1–3530.0)	2418.2 (1552.6–3324.9)	0.155	2522.3 (1670.1–3084.4)	2166.2 (1651.0–2619.9)	0.003
Potassium, mg	1779.7 (1368.4–2094.0)	1691.14 (1298.2–2075.1)	0.746	1696.5 (1255.7–2079.6)	1606.1 (1175.2–1939.2)	0.155
Calcium, mg	472.4 (323.9–607.7)	479.81 (329.05–605.8)	0.301	490.8 (334.9–604.0)	508.3 (386.6–604.5)	0.0207
Magnesium, mg	217.8 (171.2–251.0)	209.77 (151.2–256.5)	0.846	203.0 (150.0–249.1)	191.7 (142.0–223.7)	0.637
Phosphorus, mg	925.1 (720.6–1063.6)	868.8 (718.7–1036.6)	0.477	841.3 (645.9–992.2)	807.3 (662.2–924.1)	0.107
Iron, mg	13.4 (10.0–15.4)	12.4 (8.8–14.5)	0.871	12.0 (9.3–14.4)	11.1 (8.1–12.4)	0.567

**Table 4:** Vitamins of respondents.

Indicator	Male			Female		
	Mean (95%CI)		*p*-Value	Mean (95%CI)		*p*-Value
	60–74 Aged	75–90 Aged		60–74 Aged	75–90 Aged	
Retinol eq.	1107.5 (269.5–859.0)	371.8 (266.5–650.2)	0.698	761.2 (257.5–839.6)	744.4 (219.1–753.5)	0.110
Tocopherol eq.	7.6 (4.4–9.2)	6.8 (4.4–7.9)	0.897	7.0 (4.6–8.6)	6.3 (3.9–8.1)	0.159
Thiamine, mg	0.6 (0.4–0.7)	0.6 (0.3–0.8)	0.220	0.6 (0.4–0.7)	0.5 (0.4–0.62)	0.138
Riboflavin, mg	0.9 (0.6–1.0)	0.8 (0.4–1.0)	0.518	0.8 (0.5–0.9)	0.8 (0.6–0.9)	0.059
Niacin eq.	22.0 (14.8–25.1)	19.9 (13.5–25.3)	0.747	18.6 (13.1–22.4)	16.5 (13.2–21.3)	0.399
Ascorbic acid, mg	27.6 (11.7–38.1)	21.7 (9.1–29.9)	0.612	41.6 (13.6–41.7)	25.4 (10.9–35.5)	0.279

## Data Availability

The data presented in this study are available on request from the corresponding author.
